# Health Tract, No. 166

**Published:** 1863-08

**Authors:** 


### HEALTH TRACT, No. 166.

### SAYRE, THE BANKER,

Is the happiest-hearted man we ever saw! We met him yesterday in Broadway, the first time in thirty years, when we used to dine with him in the rear of his counting-room; and he didn't look a speck older than when we parted. There was the same cordial, joyous greeting; the same unbosoming of soul and self in a minute; and before we knew it, we thought it was only yesterday. Now, the main well-spring of the big Kentuckian's joyous, genial nature, and apparent defiance of years—for he weighs near two hundred, and is approaching four-score—is his most extraordinary and implicit and childlike confidence in the truth of the Christian religion, and its existence in his own heart. He never had a doubt in his life; and the faith that lives and springs perennially within him, throws sunshine, bright and beautiful, over all he casts his eye upon.

Sayre was an industrious man; he had no clerk, he clerked himself; he had no carriage, he locomoted himself, when locomotion was necessary. This was another element of his gladsome, gleesome nature, to be *always fully busy in doing something that was to purpose*, or that was pressing enough to give the feeling that it must be done, and that an advantage would come of it when completed. Take a lesson from this, ye lazy, lounging, yawning, stretching, idle folk, whose whole life is without end or aim; be busy about something useful or profitable; get out of that miserable, "ennuied" existence of yours, and human life and human kind will wear a different and a happier phase to the end of the chapter. Another reason for "Davy's" (as all his townsmen friendlily called him) happy temperament was, that he was always making money; whatever might be the sudden "stringency of the money-market," whatever the breakdowns and reverses and failures and blow-ups, he was always like a cat, sure to come down right side up, because he *never went in debt*, *never ran any risk*. What a glorious motto for our young men to begin life with; what millions of losses would it prevent; what millions of lives, worse than wasted, would it save, and crushed and ruined hearts, too! Sayre is happy and young in his old age, because he has a heart as big as all out-doors. For nearly half a century his house has been a clergyman's hotel; he has fed and lodged more ministers than any dozen men in the nation; for he and his grand, good wife were always so glad to see them, that they could not only not help going there, but they would pass the word to every "brother" who was going that way: "Put up at old Davy's." He came round to the Sunday-school one day; it was so crowded as to make it inconvenient and uncomfortable. He saw it in a moment, said not a word, but soon after took us round to a splendid new building, costing many thousand dollars, and said to us: "You haven't room enough; this is for your Sunday-school." He visited the theological seminary of his Church. Its library was in a small, cramped-up room. "Now, Humphreys," said he to the President, "we must have a separate building for this library; have it done right away; if you see that it is well done, I will pay the bills." He is happy, because, next to his religion, he loves the glorious Union. His house is the general rendezvous of all the army officers. He raised a regiment himself, and gave every man a sum of money in addition. In the course of his life, he has given five hundred thousand dollars in money, to help along various poor relations, one of whom is now at the very head of one of the learned professions. A Christian, a philanthropist, and a patriot, always temperate, always making money, we can't exactly see why he shouldn't be as happy as the day is long, and always as lively as a young kitten.

